# In Vitro Modeling of Sarcopenia-Related Pathology and Exercise-Associated Adaptations: Integration with In Silico Approaches

**DOI:** 10.3390/ijms27198609

**Published:** 2026-09-26

**Authors:** Kyung-Wan Baek, Yu Ri Jeong, Ji-Seok Kim, Ah Young Lee

**Affiliations:** 1 Research Institute of Pharmaceutical Sciences, Gyeongsang National University, Jinju 52828, Republic of Korea; baekbo1218@gmail.com (K.-W.B.); kjs7952@gnu.ac.kr (J.-S.K.); 2 Department of Physical Education, Gyeongsang National University, Jinju 52828, Republic of Korea; 3 Department of Food Science and Nutrition, Gyeongsang National University, Jinju 52828, Republic of Korea; wjddbfl5675@gmail.com

**Keywords:** sarcopenia, skeletal muscle, in vitro models, exercise-associated adaptations, engineered muscle tissue, multi-omics, machine learning

## Abstract

Sarcopenia is a progressive and generalized skeletal muscle disorder characterized primarily by reduced muscle strength, with low muscle quantity or quality confirming the diagnosis and poor physical performance indicating greater severity. Although human studies and animal models remain essential, their use is constrained by ethical considerations, inter-individual variability, species-specific differences, cost, and the difficulty of isolating individual mechanisms within whole-body systems. This review critically evaluates in vitro models that reproduce selected sarcopenia-related pathological phenotypes, experimental approaches for studying exercise-associated adaptations, and in silico methods for translational benchmarking. We compare myogenic cell cultures, three-dimensional engineered muscle tissues, senescence-inducing systems, and microphysiological platforms according to the phenotypes modeled, principal endpoints, strengths, and limitations. We then assess electrical pulse stimulation, mechanical stretch, and pathway-targeted pharmacological probes as reductionist tools for examining contraction-associated, load-sensitive, humoral, and metabolic responses rather than reproducing whole-body exercise. Finally, we discuss how multi-omics profiling, machine learning, deep learning, public transcriptomic datasets, and cell-type-aware analyses can benchmark in vitro signatures against in vivo data while accounting for batch effects, dataset heterogeneity, cell-composition confounding, overfitting, interpretability, and external validation. Integrating experimentally validated findings with clearly identified hypothesis-generating observations may improve model selection, reduce reliance on animal experiments where appropriate, and accelerate the development of interventions to preserve skeletal muscle function during aging.

## 1. Introduction

According to the EWGSOP2 (European Working Group on Sarcopenia in Older People 2) framework, sarcopenia is a progressive and generalized skeletal muscle disorder in which low muscle strength indicates probable sarcopenia, low muscle quantity or quality confirms the diagnosis, and poor physical performance denotes severe sarcopenia [1]. Although it occurs most commonly in older adults, sarcopenia may also develop earlier in life and should not be equated with loss of muscle mass alone [2]. It is associated with an increased risk of frailty, falls, disability, hospitalization, and mortality, thereby imposing a substantial socioeconomic burden on healthcare systems worldwide [3,4,5]. As populations age, clarifying the mechanisms underlying sarcopenia and developing effective preventive and therapeutic strategies are becoming increasingly important.

At the mechanistic level, the development and progression of sarcopenia involve multiple interconnected biological processes. These include an imbalance between muscle protein synthesis and degradation, mitochondrial dysfunction [6], oxidative stress [7], chronic low-grade inflammation (inflammaging) [8], impaired regenerative capacity of satellite cells [9], neuromuscular junction degradation [10], sex-hormone alterations [11], and cellular senescence [12]. These processes can converge to impair muscle quality and function; however, none of them is equivalent to sarcopenia or is sufficient by itself to constitute a complete experimental model of the disorder.

For conceptual clarity, sarcopenia should be distinguished from related but non-equivalent terms. Muscle atrophy denotes a reduction in muscle fiber or tissue size and can occur under diverse physiological or pathological conditions, whereas disuse atrophy specifically arises from reduced mechanical loading or contractile activity, such as immobilization or prolonged bed rest [13,14]. Muscle wasting is a broader descriptive term encompassing the loss of skeletal muscle mass across aging and various disease conditions [15]. Cachexia, in contrast, is a complex systemic metabolic syndrome associated with an underlying illness and characterized by the loss of muscle, with or without loss of fat mass, frequently accompanied by inflammation, anorexia, insulin resistance, and increased protein catabolism [16].

To elucidate the biological mechanisms of sarcopenia, researchers have traditionally relied on human muscle biopsies and in vivo animal models, including rodents, *Drosophila*, and zebrafish [17,18,19]. Human-derived tissues provide valuable clinical relevance but are constrained by ethical considerations, limited tissue availability, invasive sampling procedures, and substantial heterogeneity among donors [20]. Likewise, animal models require extended periods for aging phenotypes to develop, involve considerable maintenance costs, and may not fully recapitulate human physiology because of species-specific differences. Furthermore, the complexity of whole-organism systems often makes it difficult to isolate individual molecular pathways without interference from systemic and inter-organ interactions.

Given these limitations, in vitro and in silico approaches have emerged as complementary platforms rather than substitutes for human and animal studies. In vitro systems include immortalized myoblast cell lines such as C2C12 and L6 cells, primary skeletal muscle cells, and induced pluripotent stem cell (iPSC)-derived myogenic cells, as well as three-dimensional bioengineered muscle constructs [21,22,23]. These systems permit controlled investigation of individual pathological features, including myotube atrophy, oxidative stress, inflammation, mitochondrial dysfunction, and cellular senescence [24]. Catabolic and stress-related stimuli, including glucocorticoids, inflammatory cytokines, oxidative stressors, and metabolic perturbations, can therefore be used to reproduce selected sarcopenia-related phenotypes under defined experimental conditions [24,25,26]. However, these reductionist phenotypes should not be interpreted as complete models of the clinical disorder.

Beyond pathological modeling, in vitro approaches are increasingly used to investigate selected exercise-associated adaptations [27,28]. These approaches include contraction-based electrical pulse stimulation (EPS), mechanical stretch, exercise-conditioned serum or plasma, exercise-responsive myokines and exerkines, and pathway-targeted pharmacological probes. None of these approaches reproduces whole-body exercise in its entirety. Instead, each isolates a limited biological input or response, such as contractile activity, mechanical loading, circulating exercise-associated factors, or metabolic signaling. These platforms can therefore be used to investigate exercise-responsive pathways involving adenosine monophosphate-activated protein kinase (AMPK), peroxisome proliferator-activated receptor gamma coactivator 1-alpha (PGC-1α), insulin-like growth factor-1 (IGF-1)/protein kinase B (Akt)/mechanistic target of rapamycin (mTOR), calcium-dependent signaling, and mitochondrial biogenesis. Accordingly, this review uses exercise-associated adaptations as the overarching term and reserves exercise mimetic for pharmacological agents intended to activate selected exercise-responsive pathways.

Recent technological advances have expanded the capabilities of in vitro skeletal muscle models. Conventional two-dimensional (2D) cultures have been complemented by three-dimensional (3D) engineered muscle tissues (EMTs), skeletal muscle organoids, and organ-on-a-chip platforms [29,30]. Compared with 2D monolayers, these advanced platforms can better reproduce selected structural and functional features of native skeletal muscle and permit the assessment of contractile function, multicellular interactions, extracellular matrix remodeling, nutrient perfusion, and mechanical loading [30,31,32]. However, greater architectural complexity does not by itself establish a complete model of sarcopenia; the modeled phenotype, cell composition, maturation state, and validation endpoints must be explicitly defined. These systems may also enable defined pathological stimuli to be combined with exercise-associated stimulation within a single experimental framework.

In parallel, in silico approaches provide a complementary layer for interpreting and benchmarking sarcopenia-related in vitro models. Public transcriptomic and multi-omics datasets, systems-biology analyses, and machine- and deep-learning methods can be used to compare in vitro signatures with human skeletal muscle, inactivity, and exercise-response datasets and to prioritize candidate biomarkers, pathways, and interventions [33,34]. Cell-type deconvolution and single-cell reference mapping may further help distinguish myofiber-intrinsic signals from changes in tissue cellular composition [35,36].

Therefore, this review critically evaluates in vitro models used to reproduce selected sarcopenia-related pathological features, experimental approaches used to investigate exercise-associated adaptations, and in silico methods used for translational benchmarking and validation. We compare the biological questions addressed by these platforms, their principal endpoints, strengths, and limitations, as well as the extent to which their findings have been validated against human skeletal muscle data or in vivo experimental models. Particular attention is given to distinguishing experimentally validated findings from hypothesis-generating computational observations. By integrating these perspectives, this review aims to guide model selection and the interpretation of model-derived findings while identifying strategies for prioritizing candidate interventions for subsequent physiological validation. These approaches should be viewed as complements to, rather than replacements for, human and animal studies.

## 2. In Vitro Modeling of Sarcopenia-Related Pathology

### 2.1. Cell Sources and 3D Bioengineering

To determine whether exercise-associated stimulation can alleviate defined pathological features, the experimental phenotype must first be specified. Traditional two-dimensional (2D) monolayer cultures permit the controlled induction of impaired myogenic differentiation, myotube atrophy, altered proteostasis, mitochondrial dysfunction, inflammatory signaling, or cellular senescence in defined myogenic populations. Commonly used cell sources include murine C2C12 and rat L6 cell lines [37], human primary myoblasts [22], and human pluripotent stem cell (hPSC)-derived myogenic progenitors, including those generated from induced pluripotent stem cells (iPSCs) [21]. These systems differ substantially in species origin, biological fidelity, reproducibility, maturation state, and suitability for molecular or functional endpoints.

Human primary myoblasts and donor-derived iPSC models can improve human relevance and enable donor-specific analyses, but they also introduce biological and technical variability. For primary-cell studies, donor age, sex, comorbidities, medication and physical-activity history, biopsy site, isolation method, cell purity, passage number, and culture duration should therefore be reported and, where possible, balanced across comparison groups [38]. Donor age and sex can influence the expansion and metabolic characteristics of human muscle progenitor cells, while prolonged culture and repeated passaging can alter their proliferative and differentiation capacity [39,40]. For iPSC-derived systems, clone-to-clone variation and differentiation maturity must also be considered. Moreover, reprogramming through a pluripotent state can reset age-associated cellular features, potentially weakening the fidelity of iPSC-derived models intended to represent late-life sarcopenia [41].

In these systems, catabolic or aging-associated conditions are commonly induced using glucocorticoids such as dexamethasone or corticosterone [37,42]; pro-inflammatory signaling induced by tumor necrosis factor-alpha (TNF-α) [43,44,45]; oxidative stress induced by hydrogen peroxide (H_2_O_2_) [46,47]; metabolic stressors such as palmitate, amino acid deprivation, or hypoxia [7,48,49]; and senescence-associated perturbations such as budding uninhibited by benzimidazoles-related 1 (BubR1) insufficiency or doxorubicin-induced senescence and senescence-associated secretory phenotype (SASP) signaling [50,51]. Because each intervention isolates a different biological stressor, the resulting readouts should not be interpreted as interchangeable representations of sarcopenia. Rather, these systems provide experimentally tractable platforms for testing predefined phenotypes and mechanisms, as compared in Table 1 and summarized in Figure 1.

To address the structural and functional limitations of 2D monolayer cultures, three-dimensional (3D) engineered muscle tissues (EMTs) have been developed as more physiologically informative platforms for investigating skeletal muscle function and disease [30]. These systems typically embed myogenic cells within hydrogel-based matrices composed of natural extracellular matrix proteins, such as fibrin and collagen, or tissue-specific decellularized extracellular matrix (dECM) [52]. Matrix anchoring and uniaxial tension promote myotube alignment and permit the assessment of tissue-level morphology and contractile function [30]. These platforms can also be adapted to multicellular co-culture systems. In particular, the incorporation of motor neurons enables investigation of muscle-nerve interactions and functional neuromuscular junction (NMJ)-like connectivity [53]. Endothelial cells and stromal fibroblasts have also been incorporated into more specialized constructs to promote endothelial network formation, vascular organization, and perfusion-related functions [31,32]. Nevertheless, current 3D EMTs remain incompletely mature and cannot reproduce the systemic regulation, fully developed vascularization, or complex aging environment of human skeletal muscle. Accordingly, these systems should be viewed as biomimetic platforms for investigating selected aspects of skeletal muscle architecture, innervation, vascular and stromal interactions, and cell–cell communication rather than as complete replicas of aged human muscle.

### 2.2. Sustained Oxidative Stress for Modeling Senescence-Associated Phenotypes

Among oxidative stress-based approaches, bolus exposure to exogenous hydrogen peroxide (H_2_O_2_) is commonly used to induce reactive oxygen species (ROS)-mediated injury in cultured skeletal muscle cells. The biological outcome depends strongly on the concentration and duration of exposure: H_2_O_2_ can induce oxidative damage and myotube atrophy, whereas higher or prolonged exposure can activate extensive apoptotic signaling [46,54]. Consequently, this approach is better interpreted as a model of acute oxidative injury than as a complete representation of sarcopenia-associated cellular aging. By contrast, sarcopenia develops through chronic and cumulative disturbances in skeletal muscle homeostasis, including mitochondrial dysfunction, sustained redox imbalance, impaired proteostasis, persistent DNA damage responses, and cellular senescence [9,55]. These interacting processes can impair satellite cell quiescence and regenerative competence and can create an inflammatory and fibrotic niche that suppresses muscle regeneration [56,57]. Therefore, the relevance of an H_2_O_2_-based model depends on whether the selected exposure produces the predefined phenotype without excessive cytotoxicity and whether its molecular and functional features are benchmarked against aged skeletal muscle.

To provide a more sustained oxidative stimulus, Wei et al. [58] developed a hydrogen peroxide-releasing hydrogel (HRH) containing glucose oxidase (GOx), which converts glucose diffusing from the culture medium into H_2_O_2_ in a sustained and controllable manner (Figure 1). In C2C12 cultures, HRH exposure induced senescence-associated changes, including increased senescence-associated β-galactosidase activity, γH2AX accumulation, cell-cycle arrest, reduced proliferative capacity, and increased expression of p16 and p21. The platform was also adapted to a three-dimensional brain organoid model and reporter-based senolytic screening, demonstrating its versatility across different experimental formats [58]. However, it has not yet been validated in three-dimensional engineered skeletal muscle or benchmarked against aged human skeletal muscle, and its compatibility with exercise-associated stimulation remains untested. HRH should therefore be considered a proof-of-concept platform for sustained oxidative stress and selected senescence-associated phenotypes rather than a validated model of sarcopenic skeletal muscle.

**Table 1:** Comparative characteristics of in vitro systems used to model sarcopenia-related phenotypes.

Model/Cell Source	Induction or Method	Modeled Phenotype and Main Endpoints	Strengths	Limitations and Interpretation	Key References
Immortalized rodent 2D myotubes (C2C12 or L6)	Dexamethasone or corticosterone	Myotube atrophy; reduced myotube diameter; increased protein degradation; induction of atrogin-1 and MuRF1	Accessible, reproducible, and scalable; dose and exposure duration can be controlled	Species- and immortalization-related differences; primarily models acute glucocorticoid-induced catabolism rather than multifactorial aging and multicellular aging	[37,42]
Human primary myoblasts and myotubes	Isolation from skeletal muscle biopsies followed by ex vivo expansion and differentiation	Donor-associated differences in proliferation, differentiation, senescence, and metabolic responses	Greater human relevance; may retain donor-specific genetic and physiological characteristics	Influenced by donor age, sex, comorbidities, biopsy site, culture conditions, and passage number; limited expansion capacity	[39,40]
hPSC- or iPSC-derived myogenic progenitors	Directed myogenic differentiation	Myogenic differentiation, maturation, and responses to defined pathological stimuli	Renewable and scalable cell source; enables patient- and disease-specific modeling	Protocol-dependent differentiation and incomplete maturation; reprogramming may reset age-associated epigenetic and metabolic features	[41,59]
C2C12 or L6 myotubes	TNF-α exposure	Myotube atrophy; inflammatory gene-network activation; NF-κB signaling; impaired myogenic differentiation	Useful for isolating cytokine-specific mechanisms and evaluating anti-inflammatory interventions	Acute or high-dose exposure may more closely resemble inflammatory muscle wasting or cachexia than age-related sarcopenia	[43,44,45]
Differentiated C2C12 myotubes	Acute bolus H_2_O_2_ exposure	Oxidative injury; reduced myotube diameter; calpain activation; mitochondrial dysfunction; apoptosis-related signaling	Simple, rapid, and readily controlled oxidative-stress model	Produces a transient concentration spike; narrow adaptive-to-cytotoxic range; does not reproduce chronic low-grade oxidative stress	[46,47,54]
C2C12 myoblasts or myotubes	Palmitate, amino acid deprivation, or hypoxia	Insulin resistance and myotube loss; altered autophagy and mTORC1 signaling; hypoxia-associated atrophy and metabolic stress	Enables controlled investigation of nutrient- and oxygen-sensitive pathways; compatible with metabolic assays	Each stimulus represents a distinct metabolic insult and is not specific to aging or sarcopenia	[48,49,60]
BubR1-knockdown C2C12 myoblasts or C2C12 myoblasts exposed to senescent-cell-derived SASP	BubR1 knockdown or exposure to conditioned medium from doxorubicin-induced senescent cells	Impaired myogenic differentiation; increased atrogene expression; p16/p21 expression; SASP-mediated inhibition of myoblast fusion	Supports investigation of persistent senescence-associated and paracrine mechanisms	Genetic- and drug-specific effects; senescence markers are context-dependent; limited assessment of mature contractile function	[50,51]
3D EMTs with optional motor neuron, endothelial-cell, or fibroblast co-culture	Fibrin-, collagen-, or dECM-based constructs, with or without defined pathological stimuli	Myofiber alignment; force generation; calcium handling; ECM remodeling; NMJ-like connectivity; multicellular responses	Provides tissue-level architecture and functional endpoints; permits investigation of specific multicellular interactions	Higher cost and protocol variability; incomplete maturation and perfusion; lacks whole-body neural, endocrine, immune, and metabolic regulation	[30,31,32,52,53]
2D C2C12 cultures and a 3D brain organoid model	GOx-containing H_2_O_2_-releasing hydrogel	Sustained H_2_O_2_ exposure; increased SA-β-gal activity and γH2AX; cell-cycle arrest; reduced proliferation; increased p16 and p21 expression	More sustained and controllable H_2_O_2_ exposure than bolus treatment; applicable to reporter-based senolytic screening	Not validated in 3D engineered skeletal muscle or against aged human skeletal muscle; not specific to sarcopenia; biomaterial and diffusion effects require appropriate controls	[58]

Abbreviations: 2D, two-dimensional; 3D, three-dimensional; BubR1, budding uninhibited by benzimidazoles-related 1; dECM, decellularized extracellular matrix; ECM, extracellular matrix; EMT, engineered muscle tissue; GOx, glucose oxidase; H_2_O_2_, hydrogen peroxide; hPSC, human pluripotent stem cell; iPSC, induced pluripotent stem cell; mTORC1, mechanistic target of rapamycin complex 1; MuRF1, muscle RING finger-1; NF-κB, nuclear factor kappa B; NMJ, neuromuscular junction; SA-β-gal, senescence-associated β-galactosidase; SASP, senescence-associated secretory phenotype; TNF-α, tumor necrosis factor alpha; γH2AX, phosphorylated histone H2AX. Each system reproduces selected sarcopenia-related phenotypes and should not be interpreted as a complete model of sarcopenia.

## 3. In Vitro Modeling of Exercise-Associated Adaptations and Omics Analysis

### 3.1. Electrical Pulse Stimulation as a Contraction-Based Model

Electrical pulse stimulation, often termed exercise-like electrical pulse stimulation (EL-EPS), is a contraction-based in vitro approach used to reproduce selected cellular consequences of skeletal muscle contractile activity. Programmable electrical pulses delivered through culture-compatible electrodes induce membrane depolarization, intracellular Ca^2+^ cycling, and repetitive contractile activity in differentiated myotubes without requiring intact motor neuron input [61,62]. Because the resulting responses depend strongly on stimulation parameters, including voltage, pulse width, frequency, duty cycle, total stimulation duration, and the maturation state of the culture, protocol selection should be linked to prespecified contractile, metabolic, or transcriptional endpoints rather than interpreted broadly as a direct in vitro equivalent of endurance or resistance exercise [61,62].

In 2D myotube cultures, EL-EPS is particularly useful for examining contraction-induced molecular and metabolic responses. Studies using human and murine myotubes have reported activation of Ca^2+^-dependent and AMPK-associated signaling, increased glucose uptake and fatty acid oxidation, and transcriptional changes related to mitochondrial and myofibrillar remodeling [61,62,63]. In cultured human skeletal muscle cells, contractile activity has also been shown to prevent experimentally induced insulin resistance by suppressing pro-inflammatory signaling [64]. These findings indicate that EL-EPS can be used to determine whether pathological myotubes retain the capacity to generate selected exercise-associated adaptations. However, because 2D monolayers lack tissue-level alignment, passive tension, and direct force transmission, they are better suited for molecular, transcriptional, and metabolic endpoints than for evaluating integrated contractile physiology.

In 3D EMTs, electrical stimulation can be applied to aligned, matrix-anchored tissues, allowing repeated contraction to be induced and assessed within a tissue-like architecture. Compared with 2D cultures, these systems permit the simultaneous evaluation of molecular and metabolic adaptations and functional outcomes, including calcium transients, twitch or tetanic force, and fatigue-related responses [30]. In tissue-engineered human skeletal muscle, one week of intermittent electrical stimulation increased myobundle size, sarcomeric protein abundance, calcium-transient amplitude, tetanic force, and metabolic flux, whereas fatigue resistance was reduced relative to unstimulated controls [65]. These findings demonstrate that the effects of electrical stimulation depend on the stimulation protocol and the selected endpoint. Nevertheless, electrically stimulated 3D EMTs should be interpreted as contraction-based models rather than direct in vitro equivalents of endurance or resistance exercise because they lack systemic neuroendocrine regulation, vascular perfusion, immune-cell interactions, substrate delivery, and the complex loading patterns present in vivo.

Coupling EL-EPS with transcriptomic, proteomic, metabolomic, and secretomic analyses extends this platform beyond contraction assays by enabling the identification of contraction-responsive molecular signals [66]. At the experimental level, electrical stimulation of cultured muscle cells has reproduced selected transcriptional changes observed in trained mouse skeletal muscle and identified contraction-inducible CXC chemokines [63,67]. However, candidates identified in these reductionist systems should not automatically be classified as physiologically relevant exerkines, because systemic exercise responses include proteins, metabolites, nucleic acids, and extracellular vesicle-associated cargo originating from multiple tissues [66,68,69]. In vitro secretome profiles are also sensitive to culture maturity, medium composition, stimulation parameters, cell viability, and contamination by intracellular proteins released from damaged or dying cells [70]. Extracellular vesicle studies additionally require standardized nomenclature, separation, characterization, and reporting in accordance with current MISEV guidelines [71]. Therefore, EL-EPS-based omics analyses should be regarded primarily as hypothesis-generating approaches. Candidate mediators require independent validation in human exercise samples or animal exercise models, orthogonal molecular assays, and targeted loss- or gain-of-function experiments before their physiological or causal relevance can be established [66,72].

### 3.2. Mechanical Stretch

Mechanical stretch complements contraction-based EL-EPS by isolating the deformation-sensitive component of skeletal muscle loading. In these systems, myogenic cells or EMTs are cultured on deformable membranes, elastic substrates, or anchored hydrogel constructs and exposed to static or cyclic strain defined by its amplitude, frequency, direction, and duration [30,73]. Unlike EL-EPS, which induces membrane depolarization and repetitive contractile activity, mechanical stretch primarily probes how muscle cells sense and respond to deformation through the extracellular matrix, focal adhesions, cytoskeleton, sarcolemma-associated mechanosensors, and sarcomeric structures. Accordingly, mechanical stretch should be interpreted as a reductionist mechanobiological platform for investigating load-sensitive signaling rather than as a complete in vitro surrogate for resistance exercise.

The biological effects of mechanical stretch depend on strain magnitude, frequency, duration, and culture geometry, including tissue maturity and matrix anchoring. In primary chick skeletal myotubes, 72 h of passive cyclic stretch induced hypertrophy through PI3K/Akt/TOR signaling, as inhibition of PI3K or mTOR largely suppressed the hypertrophic response [74]. Mechanical loading has also been integrated directly with pathological induction in C2C12-derived 3D engineered muscle. Loading applied either before or after dexamethasone exposure attenuated myotube atrophy and functional deterioration and prevented dexamethasone-induced increases in MuRF-1 (*Trim63* gene) and MAFbx (*Fbxo32* gene) mRNA expression [75]. Together, these findings support the use of mechanical loading as a mechanistically defined intervention for testing load-associated protective responses in vitro. Nevertheless, these effects cannot be generalized across static, cyclic, uniaxial, multiaxial, and overload regimens; loading parameters and viability or injury controls should therefore be reported explicitly [30].

Mechanical loading is relevant to sarcopenia research because aging can alter both the anabolic response to resistance exercise and the mechanical microenvironment of skeletal muscle. In older men, the dose–response relationships of myofibrillar protein synthesis and the phosphorylation of p70S6K and 4E-BP1 following resistance exercise were blunted compared with those in younger men [76]. Separately, the extracellular matrix of aged skeletal muscle exhibits increased stiffness and can redirect muscle stem cell fate toward a fibrogenic phenotype [77]. These findings provide a rationale for applying standardized stretch protocols to aged donor-derived cells or cultures exhibiting defined sarcopenia-related phenotypes to determine whether their responses to mechanical input are preserved or impaired. Such studies should compare pathologically challenged and control cultures under identical loading conditions and incorporate both molecular and functional endpoints. Transcriptomic, proteomic, phosphoproteomic, or secretomic profiling may identify candidate load-responsive pathways, but these results should be considered hypothesis-generating until independently replicated and functionally validated.

Mechanical stretch should therefore be interpreted as a load-associated stimulus rather than an in vitro reproduction of resistance exercise. In vivo resistance exercise integrates active contraction, external loading, motor-unit recruitment, muscle-tendon force transmission, vascular perfusion, and systemic endocrine and immune responses, whereas mechanical stretch isolates primarily the deformation-sensitive component of mechanotransduction. Direct comparison with human resistance-exercise responses therefore requires explicit benchmarking against appropriate in vivo datasets and, when feasible, parallel comparison with contraction-based stimulation. Within these boundaries, mechanical stretch complements EL-EPS by enabling controlled investigation of how tensile strain influences tissue remodeling, anabolic signaling, and stress-adaptive responses [30].

### 3.3. Pathway-Targeted Pharmacological Exercise Mimetics

Pharmacological exercise mimetics are compounds intended to activate selected molecular pathways associated with exercise adaptation. Because they do not reproduce contractile activity, mechanical loading, or systemic interorgan signaling, their use in vitro should be interpreted as a pathway-targeted perturbation rather than as a complete model of exercise. Major targets include AMP-activated protein kinase (AMPK), peroxisome proliferator-activated receptor delta (PPARδ), calcium-dependent signaling, cyclic adenosine monophosphate (cAMP)/protein kinase A (PKA) signaling, and oxidative metabolic regulation [78,79]. In cultures exhibiting defined sarcopenia-related phenotypes, these agents can be used to determine whether specific exercise-responsive pathways remain inducible and whether their activation modifies relevant molecular or functional endpoints. However, activation of an isolated signaling pathway should not, by itself, be considered evidence of an exercise-associated adaptation unless it is accompanied by relevant downstream phenotypic changes and benchmarked against contraction-based stimulation or in vivo exercise responses.

5-Aminoimidazole-4-carboxamide ribonucleoside (AICAR; also known as AICA riboside) is one of the most widely used pharmacological activators of AMPK-related metabolic signaling. Following cellular uptake, AICAR is phosphorylated to 5-aminoimidazole-4-carboxamide ribonucleotide (ZMP), an AMP analogue that activates AMPK. In rat skeletal muscle, AICAR increased AMPK activity, fatty acid oxidation, and glucose uptake [80]. Direct evidence is also available from skeletal muscle cell culture: in C2C12 myotubes, AICAR treatment increased AMPK activation, mitochondrial content, peak mitochondrial respiratory capacity, and the expression of regulators associated with mitochondrial biogenesis, while reducing glycolytic metabolism [81]. These findings support the use of AICAR to investigate AMPK-related metabolic remodeling in vitro. However, AICAR does not reproduce the mechanical, calcium-dependent, neural, endocrine, or immune components of endurance exercise. AICAR-induced responses should therefore be described as pharmacological activation of AMPK-related metabolic pathways rather than as a complete in vitro model of endurance exercise.

Calcium-mobilizing compounds provide another pathway-targeted approach for investigating exercise-responsive signaling. In noncontracting L6 myotubes, intermittent exposure to ionomycin or caffeine for 5 h per day increased mitochondrial enzyme levels. In contrast, continuous ionomycin exposure for 5 days caused an approximately 40% reduction in protein per culture dish, indicating substantial myotube loss [82]. This protocol-dependent contrast suggests that intermittent increases in cytosolic Ca^2+^ can promote mitochondrial remodeling, whereas sustained Ca^2+^ elevation may produce cellular damage rather than an adaptive response. Supporting the biological relevance of this pathway, skeletal muscle-specific expression of constitutively active CaMKIV in transgenic mice increased mitochondrial DNA replication, mitochondrial biogenesis, mitochondrial enzyme expression, and PGC-1 expression [83]. However, this evidence was obtained from a transgenic in vivo model and should not be presented as direct validation of caffeine- or ionomycin-treated muscle cultures. Therefore, caffeine and ionomycin are best used as Ca^2+^-mobilizing mechanistic probes rather than as stand-alone exercise surrogates, with exposure schedule, cell viability, intracellular Ca^2+^ responses, and mitochondrial endpoints reported explicitly.

Additional pharmacological compounds target discrete metabolic or second-messenger pathways but should not be grouped uncritically as exercise mimetics. Forskolin directly activates adenylyl cyclase and elevates intracellular cAMP, thereby allowing cAMP/PKA/CREB signaling to be investigated while bypassing adrenergic receptor activation. In C2C12 myotubes, forskolin increased CREB-PGC-1α interaction and the mRNA expression of *Fndc5* and *Ppargc1a* [84]. The selective PPARβ/δ agonist GW501516 increased the expression of genes involved in preferential lipid utilization, β-oxidation, cholesterol efflux, and energy uncoupling in skeletal muscle cells [85]. In contrast, palmitate should be classified as a lipotoxic pathological stressor rather than as an exercise mimetic; in C2C12 myotubes, palmitate induced insulin resistance and myotube loss and reduced the mRNA expression of *Fndc5*, *Erfe*, and *Fgf21* [48]. Combining palmitate exposure with cAMP- or Ca^2+^-mobilizing compounds may therefore be useful for testing whether pathway-targeted interventions rescue defined metabolic abnormalities. However, such experiments should be described as a pathological challenge combined with a pathway-targeted intervention, rather than as an exercise-mimicking cocktail, unless the resulting responses are benchmarked against contraction-based stimulation and in vivo exercise datasets.

Accordingly, pharmacological exercise mimetics are best positioned as mechanistic probes rather than as stand-alone exercise surrogates. In models exhibiting defined sarcopenia-related phenotypes, these compounds can be used to determine whether AMPK-, Ca^2+^-, cAMP/PKA-, or PPARβ/δ-dependent responses remain inducible. Evidence of a protective effect should require improvement in a prespecified pathological endpoint, such as myotube diameter, contractile force, mitochondrial respiration, insulin responsiveness, or proteostasis, rather than activation of the targeted signaling pathway alone. Dose–response relationships, exposure duration, vehicle controls, and cytotoxicity should also be reported to distinguish adaptive signaling from nonspecific cellular stress. Where possible, pharmacological responses should be compared with EL-EPS or mechanical loading and benchmarked against relevant in vivo exercise datasets. The principal biological inputs, response endpoints, strengths, and interpretive limitations of these in vitro approaches are compared in Table 2. Omics analyses can support these comparisons and identify candidate mechanisms, but such candidates should be considered hypothesis-generating until independently replicated and functionally validated (Figure 2).

## 4. In Silico Predictive Modeling, Machine Learning Classifiers, and Cell-Type-Aware Candidate Biomarker Prioritization

In silico analyses can provide a computational bridge between reductionist models of exercise-associated adaptations and the molecular complexity of exercise in vivo. Because EL-EPS, mechanical stretch, and pharmacological exercise mimetics capture only selected biological inputs, their physiological relevance cannot be established from the induction of a few canonical markers alone. Instead, molecular profiles generated in vitro should be compared with independent human and animal exercise datasets at the gene, pathway, and cell-type levels. Transcriptomic integration, dimensionality reduction, machine learning, and cell-type-aware analyses can then be used to quantify cross-dataset concordance, identify signals consistently retained across experimental contexts, and prioritize candidate biomarkers or mechanisms for further testing [33,34,88,89]. These computational outputs should be regarded as hypothesis-generating unless they are reproduced in independent cohorts and supported by targeted experimental and functional validation (Figure 2).

### 4.1. Deep Learning-Based Benchmarking of In Vitro Exercise-Associated Adaptations and Candidate Myokine Prioritization

Lee et al. provided a representative example of deep learning-based cross-dataset benchmarking of an in vitro contraction model [33]. Differentiated C2C12 myotubes were subjected to EL-EPS for 24 h, and RNA sequencing identified 774 differentially expressed genes, comprising 406 upregulated and 368 downregulated genes, relative to unstimulated controls [33]. These genes were used to construct a Deep Belief Network (DBN) classifier, which was subsequently applied to nine publicly available skeletal muscle exercise datasets comprising 108 samples. The classifier assigned 50 of 60 sedentary samples (83%) to the control class and 32 of 48 exercised samples (67%) to the EPS class [33]. These findings provide cross-dataset computational evidence that EL-EPS retains a measurable subset of the transcriptional response to exercise in vivo. However, because the model was derived from a small discovery experiment comprising three control and four EPS samples, these results should not be interpreted as independent biological validation of EL-EPS as a whole-body exercise surrogate.

The principal value of this analysis lies not in demonstrating that EL-EPS reproduces whole-body exercise, but in delineating the transcriptional programs that can be elicited within contracting myotubes. EL-EPS isolates myotube-intrinsic and autocrine responses while excluding major components of exercise in vivo, including neural recruitment, vascular perfusion, systemic substrate delivery, endocrine and immune responses, and inter-organ communication [33]. Accordingly, genes showing concordant directional changes across EL-EPS and exercise datasets may be prioritized as candidate contraction-associated responses. Conversely, discordant genes should not automatically be attributed to systemic exercise inputs, because differences in species, muscle group, exercise modality and intensity, sampling time, participant phenotype, analytical platform, data normalization, and tissue cellular composition may also contribute [34]. Cross-dataset concordance should therefore be interpreted as a framework for prioritizing mechanisms and follow-up experiments rather than as proof that EL-EPS reproduces exercise in vivo.

Computational benchmarking can also support the prioritization of candidate myokines. Lee et al. screened EPS-responsive genes annotated as encoding extracellular proteins and identified amphiregulin, an epidermal growth factor receptor ligand encoded by *Areg*, as a candidate exercise-responsive myokine [33]. In C2C12 myotubes, EL-EPS increased *Areg* mRNA expression and AREG protein abundance in conditioned medium, while *Areg* expression was also elevated in murine skeletal muscle after acute maximal exercise [33]. These findings provide transcript-level, secretory, and in vivo expression concordance, but they do not demonstrate that muscle-derived AREG is necessary or sufficient for exercise adaptation. Burzyn et al. showed that regulatory T-cell-derived AREG acted directly on muscle satellite cells in vitro and improved muscle repair after injury in mice [90]. This finding supports the biological plausibility of AREG in muscle regeneration, but it represents a different cellular source and injury context. Accordingly, AREG should be considered a candidate exercise-responsive myokine with potential relevance to sarcopenia rather than a validated sarcopenia-directed therapeutic target. Further validation would require identification of its cellular source and target cells in aged exercising muscle, loss- and gain-of-function studies, and demonstration of improvements in regenerative or contractile outcomes.

### 4.2. Machine Learning Classification of Age- and Exercise-Associated Skeletal Muscle Transcriptomic States

Machine learning can also be applied to public human skeletal muscle transcriptomic datasets to examine age- and exercise-associated molecular states. This approach is conceptually distinct from the DBN-based EL-EPS model described above. Rather than testing whether an in vitro contraction signature classifies exercise responses in vivo, it reanalyzes existing human datasets to identify transcriptomic features that distinguish age, inactivity, and training status. Public repositories such as the National Center for Biotechnology Information Gene Expression Omnibus (GEO) facilitate this approach by providing transcriptomic datasets and associated study metadata. Dimensionality reduction, supervised classification, and decision tree modeling can then be used to explore candidate classifier features [89]. However, because skeletal muscle transcriptomic datasets frequently contain far more molecular features than biological samples, exploratory classification is particularly susceptible to overfitting, dataset-specific effects, and confounding by study design. Consequently, features identified from a single public dataset should be regarded as hypothesis-generating rather than as validated biomarkers.

Baek et al. presented illustrative reanalyses of two public skeletal muscle transcriptomic datasets [89]. GSE8479 was originally generated to compare skeletal muscle from young adults and older adults before and after resistance training [91]. Principal component analysis, logistic regression, and decision tree modeling of this dataset identified *USP54* and *ATP1B4* as candidate features that could contribute to the classification of age- and training-associated transcriptomic states [89]. A similar exploratory analysis was performed using GSE28422, which contains skeletal muscle transcriptomic data from young and older adults exposed to acute resistance exercise and resistance training [92]. In this analysis, *DMRT2* and *PRND* were identified as candidate decision tree features [89]. These analyses demonstrate how machine learning can generate testable candidates from previously collected exercise datasets, but they do not establish these genes as biomarkers of sarcopenia or skeletal muscle rejuvenation. The analyses were based on individual public datasets and lacked independent external validation and functional confirmation. Accordingly, *USP54*, *ATP1B4*, *DMRT2*, and *PRND* should be considered illustrative candidate classifier features until their reproducibility is demonstrated across independent cohorts and their biological relevance is confirmed at the transcript, protein, cellular, and functional levels.

### 4.3. Cell-Type-Aware Interpretation of Bulk Transcriptomics and Single-Cell Reference Mapping

Large-scale skeletal muscle transcriptomic resources are valuable for identifying exercise- and inactivity-associated molecular signatures. MetaMEx integrates 66 published human skeletal muscle transcriptomic datasets representing more than 1100 individuals and enables responses to be examined across exercise modality, acute exercise versus training, participant phenotype, and sampling-related variables [34]. Its scale permits candidate exercise-responsive genes to be evaluated across multiple studies rather than inferred from a single cohort. However, most contributing datasets were generated from bulk muscle biopsies, and the resulting expression values represent mixtures of signals from multiple cell populations. Consequently, replication across bulk datasets does not by itself establish that a response is intrinsic to myofibers.

This limitation is particularly important in aging and sarcopenia, in which muscle biopsies may differ in myofiber composition and in the relative abundance or activation state of satellite cells, fibro-adipogenic progenitors, endothelial cells, immune cells, and other stromal populations. A change in bulk expression may therefore reflect altered transcription within a stable cell population, a change in cellular abundance, or both. Inflammatory infiltration, vascular remodeling, fibrosis, and shifts in fiber-type composition can consequently produce apparent age- or exercise-associated signals that are not exclusively myofiber-intrinsic. Candidate biomarkers derived from bulk data should therefore be interpreted as tissue-level signals unless their cellular source has been independently established [88,93,94].

Cell-type expression resources can help refine this interpretation. Comparative analyses suggest that transcripts such as *PPARGC1A*, *AKT1*, *SLC2A4*, *MAPK1*, and *COX4I1* are relatively enriched in skeletal muscle or myogenic compartments, whereas transcripts such as *MTOR* and *RPS6KB1* may also be contributed by non-myogenic cell populations [88]. This information can increase confidence in the likely cellular origin of a bulk signal, but enrichment alone does not establish that a gene mediates exercise adaptation. Cell-type localization, differential expression, protein abundance, and functional relevance remain separate levels of evidence.

Reference-guided deconvolution provides a more systematic approach. CIBERSORTx can estimate cell-type abundance and infer cell-type-specific expression from bulk profiles, whereas MuSiC uses multi-subject single-cell reference data to estimate cellular proportions [35,36]. Skeletal muscle single-cell datasets have further characterized muscle stem cells, fibro-adipogenic progenitors, endothelial cells, immune cells, and other resident populations that can serve as reference profiles [93,94].

Cell-type-aware interpretation also informs the selection of in vitro models. When a candidate response is consistently localized to the myogenic compartment, EL-EPS-treated myotubes or pathway-targeted pharmacological models may be suitable for mechanistic testing. When the response depends on endothelial–myogenic communication, immune–stromal signaling, fibro-adipogenic remodeling, or satellite cell–niche interactions, a myotube monoculture is unlikely to capture the complete mechanism. In such cases, 3D engineered muscle tissues, co-culture systems, organ-on-chip platforms, or other microphysiological models may be more appropriate. Cell-type mapping can therefore guide experimental model selection, but it cannot substitute for targeted functional validation.

### 4.4. Methodological Limitations and a Tiered Validation Framework

Cross-study transcriptomic integration is vulnerable to technical batch effects and biological heterogeneity arising from differences in analytical platforms, laboratories, participant characteristics, muscle groups, exercise modalities, biopsy timing, and preprocessing procedures. Batch-adjustment methods such as ComBat can reduce unwanted technical variation and facilitate cross-dataset comparisons [95]. However, when biological groups are unevenly distributed across batches or the phenotype of interest is confounded with batch, correction may attenuate genuine biological differences or produce excessive confidence in downstream results [96]. Batch correction should therefore be accompanied by an examination of study design and data distributions before and after adjustment and should not be assumed to make biologically heterogeneous datasets directly equivalent.

Overfitting represents a further concern when thousands of molecular features are analyzed in cohorts containing relatively few samples. All data-dependent procedures, including normalization, batch adjustment, feature selection, and hyperparameter tuning, should be performed within the training data of each validation fold to prevent information leakage. When model selection and performance evaluation are conducted using the same cross-validation procedure, prediction accuracy can be substantially overestimated; nested cross-validation provides a less biased estimate of generalizability [97]. Nevertheless, internal cross-validation cannot replace testing in a genuinely independent cohort. When multiple studies are available, leave-one-study-out validation can further determine whether classifier performance generalizes across cohorts rather than reflecting study-specific characteristics. Performance should therefore be reported using multiple measures, including discrimination, uncertainty, and class-specific performance, rather than a single accuracy value. Model interpretability must also be considered separately from predictive performance. A classifier may distinguish experimental groups accurately while relying on features associated with technical artifacts, cellular composition, or cohort-specific characteristics rather than biologically meaningful mechanisms. As discussed in Section 4.3, cell-type deconvolution and single-cell reference mapping can reduce ambiguity arising from bulk transcriptomics, but they remain dependent on the quality and compatibility of the reference data [35,36,93,94]. Interpretable pathway-level analyses, parsimonious feature sets, and examination of feature stability across resampling procedures can help determine whether model predictions are supported by reproducible biology. These computational analyses should be used to prioritize candidate mechanisms for subsequent testing rather than being interpreted as evidence of causality.

For the purposes of this review, computational findings can be organized using a four-tier validation framework. Level 1 comprises discovery within a single dataset and should be regarded as hypothesis-generating. Level 2 requires replication in independent datasets or across analytical platforms, together with concordant directionality and biologically plausible cell-type localization. Level 3 requires targeted experimental confirmation in an appropriate in vitro system, including transcript- or protein-level measurement and perturbation of the proposed mechanism. Level 4 requires physiological validation in animal models or human cohorts using functional endpoints relevant to sarcopenia. Under this framework, *USP54*, *ATP1B4*, *DMRT2*, and *PRND* remain Level 1 candidate markers because they were nominated through secondary computational analyses without independent experimental validation [89]. AREG has additional cross-context and protein-level support but has not yet been causally validated as a sarcopenia-specific mediator of exercise-associated adaptation [33,90].

Atomistic and structure-based computational approaches, including molecular docking and molecular dynamics simulations, may complement transcriptomic and machine-learning analyses by evaluating the binding modes, stability, and target selectivity of candidate pathway-targeted exercise mimetics. However, these approaches operate at the molecular-interaction level and cannot by themselves establish equivalence to exercise or therapeutic efficacy in sarcopenia-related systems. Their predictions therefore require subsequent biochemical target-engagement assays and validation in cell-based, engineered muscle, animal, and human models.

Although applications directly focused on sarcopenia remain limited, molecular modeling can bridge pathway-level observations and experimentally testable molecular mechanisms. For example, virtual screening, molecular docking, protein–protein interaction modeling, and molecular dynamics simulations have been used to evaluate dithymoquinone as a putative myostatin inhibitor and to predict its effects on the interaction between myostatin and activin receptor type-2B [98]. In a complementary study integrating computational and experimental approaches, molecular dynamics simulations combined with C2C12 myoblast assays showed that Ser46 phosphorylation altered the conformational stability and nuclear localization of sirtuin 1 and was associated with mitochondrial dysfunction, nuclear factor kappa-light-chain-enhancer of activated B cells activation, and senescence-related responses [99]. Similar workflows could be applied to prioritize ligands, post-translational modifications, binding sites, and protein–protein interfaces involving other sarcopenia-relevant targets and to generate specific hypotheses for testing in the cell-based and engineered muscle platforms discussed in this review. Molecular modeling may therefore provide a mechanistic bridge between omics- or pathway-level discovery and targeted experimental validation, although its application to sarcopenia remains at an early stage.

## 5. Conclusions and Future Perspectives

### 5.1. Conclusions

Sarcopenia research is moving beyond descriptive accounts of age-related muscle decline toward experimental systems that investigate how defined pathological and exercise-responsive signals influence skeletal muscle aging. Human and animal studies remain indispensable for establishing physiological and clinical relevance, but ethical constraints, cost, inter-individual variability, species differences, and whole-body complexity can limit the isolation of individual mechanisms [17,18,19,20]. In vitro and in silico platforms should therefore be viewed as complementary systems for investigating sarcopenia-related phenotypes and exercise-associated adaptations under controlled conditions, rather than as substitutes for organismal models or as complete models of sarcopenia.

A major strength of contemporary in vitro modeling is its ability to separate, combine, and compare distinct dimensions of skeletal muscle biology. Defined pathological stimuli can reproduce selected cellular features relevant to sarcopenia, including myotube atrophy, mitochondrial dysfunction, inflammatory signaling, impaired proteostasis, and cellular senescence. In parallel, EL-EPS, mechanical stretch, and pathway-targeted pharmacological probes provide reductionist tools for investigating contraction-associated, load-sensitive, and metabolic signaling pathways [30,58,61,62,73,79,80]. Table 1 and Table 2 comparatively summarize the modeled phenotypes, major endpoints, strengths, limitations, and appropriate interpretation of each experimental approach. Three-dimensional EMTs, neuromuscular co-cultures, vascularized constructs, and microphysiological systems further extend this framework by incorporating tissue architecture, force transmission, cell–cell communication, and extracellular matrix remodeling [30,31,32,52,53]. Although none of these systems fully reproduces whole-body exercise or the complexity of aged skeletal muscle, together they enable experimental models to be selected according to the specific biological question.

The value of these platforms is further strengthened when multi-omics profiling is coupled with rigorous computational analysis. Transcriptomics, proteomics, secretomics, metabolomics, and extracellular vesicle profiling can identify candidate pathways, biomarkers, and myokine-like mediators. Deep learning, machine learning, cross-dataset comparison, and cell-type-aware analysis can then assess whether these signatures are reproducible across independent exercise datasets and whether bulk-tissue signals plausibly arise from myofibers or non-myofiber cellular compartments [33,34,88,89]. The tiered validation framework outlined in Section 4.4 clarifies the corresponding levels of evidence: computational discovery generates hypotheses, independent replication strengthens confidence in their reproducibility, and experimental perturbation followed by physiological validation is required to establish biological relevance. Accordingly, in silico analysis should be regarded as a tool for interpretation and candidate prioritization rather than as a substitute for experimental validation.

### 5.2. Future Perspectives

Future progress will depend on the development of models that are both physiologically informative and rigorously benchmarked. Patient-derived iPSC-based myogenic systems, 3D EMTs, organ-on-chip platforms, and multicellular co-cultures may allow investigators to examine how genetic background, aging-related stress, exercise-associated stimulation, and intercellular communication interact within a defined experimental setting [31,32,59]. These platforms should be compared with animal exercise models, human exercise datasets, single-cell reference maps, and functional endpoints such as contractility, regenerative capacity, proteostasis, and metabolic resilience. Such benchmarking will be essential for distinguishing reproducible sarcopenia-related mechanisms from culture- or dataset-specific artifacts.

Interpretation of human primary myoblast and iPSC-derived models requires particular caution. Primary myoblasts may retain donor-specific biological characteristics, but their proliferative and differentiation capacities can vary according to donor age, sex, body mass index, previous medical treatments, and the anatomical site from which the muscle sample was obtained [100]. Comorbidities, medications, physical activity, cell purity, passage number, and cumulative culture duration should therefore be reported and controlled whenever possible. Repeated expansion can alter the proliferative and metabolic characteristics of primary myoblasts, emphasizing the importance of passage-matched experiments and the inclusion of cells from multiple independent donors [101].

Patient-derived iPSCs preserve the donor’s genetic background, but reprogramming through a pluripotent state can reset or attenuate age-associated epigenetic and transcriptomic characteristics [102]. Consequently, iPSCs generated from an older donor should not automatically be assumed to produce an aged skeletal muscle phenotype after myogenic differentiation. Studies using iPSC-derived myogenic cells should employ multiple clones, standardized differentiation and maturation procedures, and direct assessment of relevant aging hallmarks. Comparison with donor-derived primary cells or the application of defined pro-aging stimuli may also be necessary to determine which aspects of skeletal muscle aging are retained or experimentally reconstructed.

An important future direction is to align model complexity with the biological question being investigated. For pathways that are primarily myofiber-intrinsic, reductionist systems such as EL-EPS-treated myotubes, mechanically stretched myotubes, or pathway-targeted pharmacological models may be sufficient for mechanistic testing. In contrast, pathways involving immune–stromal remodeling, endothelial–myogenic communication, neuromuscular interactions, or satellite cell–niche regulation will require more complex platforms, including 3D co-cultures, vascularized constructs, innervated muscle models, or microphysiological systems. This mechanism-matched model-selection strategy will be essential for determining which findings can be translated from controlled in vitro systems to the multicellular pathology of sarcopenia.

The integration of pathological in vitro modeling, exercise-associated stimulation, multi-omics profiling, and in silico analysis provides a complementary framework for sarcopenia research. These approaches are consistent with the 3R principles by reducing reliance on animal experimentation where appropriate while enabling mechanistic screening of pathway-targeted pharmacological candidates, senotherapeutics, regenerative factors, and myokine-like mediators [22,27]. Their greatest value lies not in replacing human or animal studies, but in refining the hypotheses and experimental priorities that these studies should address. By linking controlled experimental modeling with computational prioritization, independent replication, mechanistic perturbation, and physiological validation, next-generation in vitro and in silico platforms may contribute to the development of targeted strategies for preserving skeletal muscle function during aging.

## Figures and Tables

**Figure 1:**
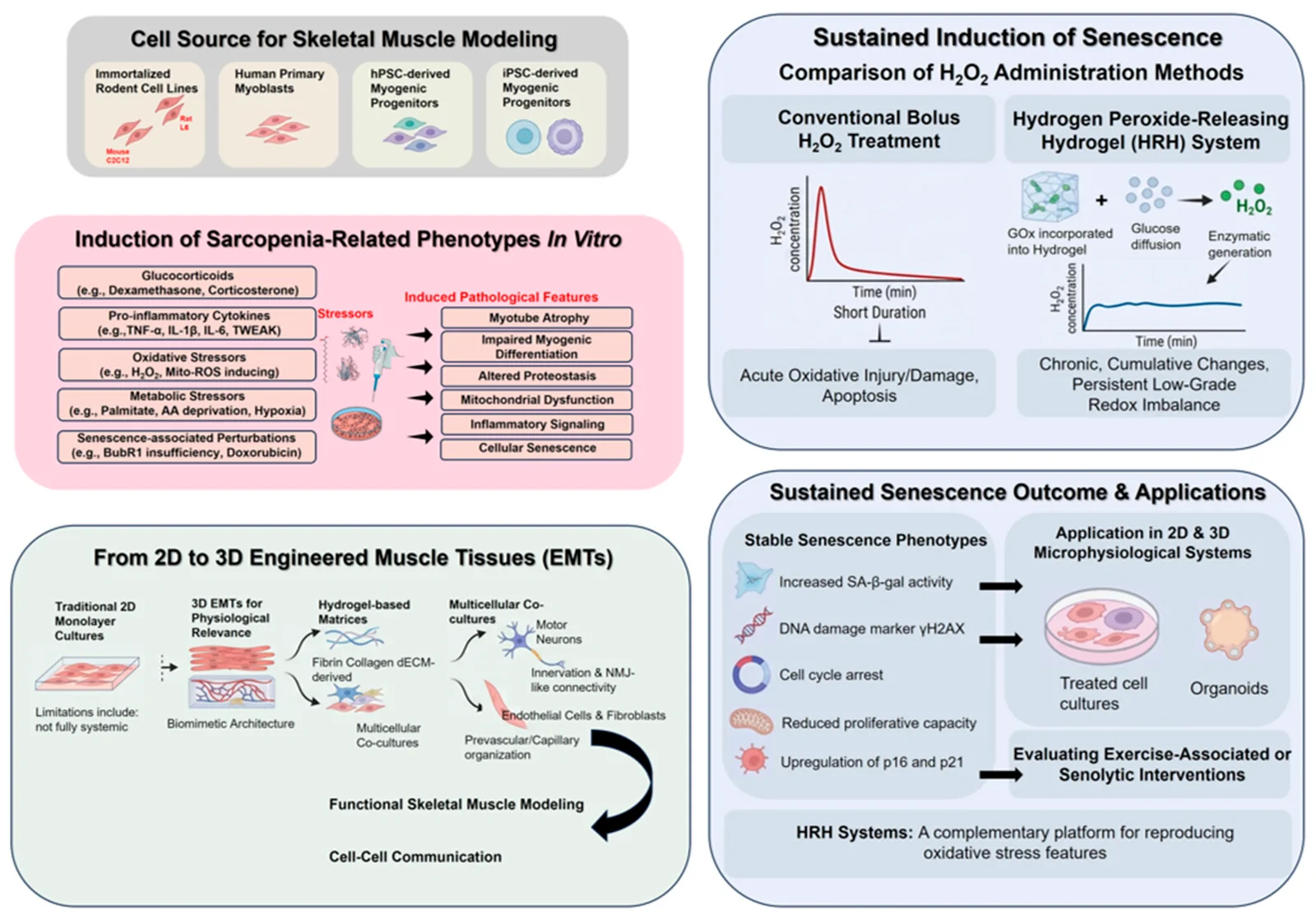
Overview of in vitro pathological modeling strategies for sarcopenia-related phenotypes. The framework includes the selection of suitable cell sources and the application of diverse pathological stressors to induce selected sarcopenia-related phenotypes. Conventional 2D monolayer cultures can be advanced to 3D engineered muscle tissues (EMTs) incorporating biomimetic matrices and multicellular co-cultures. The figure also compares conventional bolus H_2_O_2_ treatment with hydrogen peroxide-releasing hydrogel (HRH) systems for inducing sustained oxidative stress and senescence-associated phenotypes. AA, amino acid; BubR1, budding uninhibited by benzimidazoles-related 1; dECM, decellularized extracellular matrix; EMT, engineered muscle tissue; GOx, glucose oxidase; hPSC, human pluripotent stem cell; HRH, hydrogen peroxide-releasing hydrogel; iPSC, induced pluripotent stem cell; Mito-ROS, mitochondrial reactive oxygen species; NMJ, neuromuscular junction; SA-β-gal, senescence-associated β-galactosidase; TNF-α, tumor necrosis factor alpha; TWEAK, tumor necrosis factor-like weak inducer of apoptosis.

**Figure 2:**
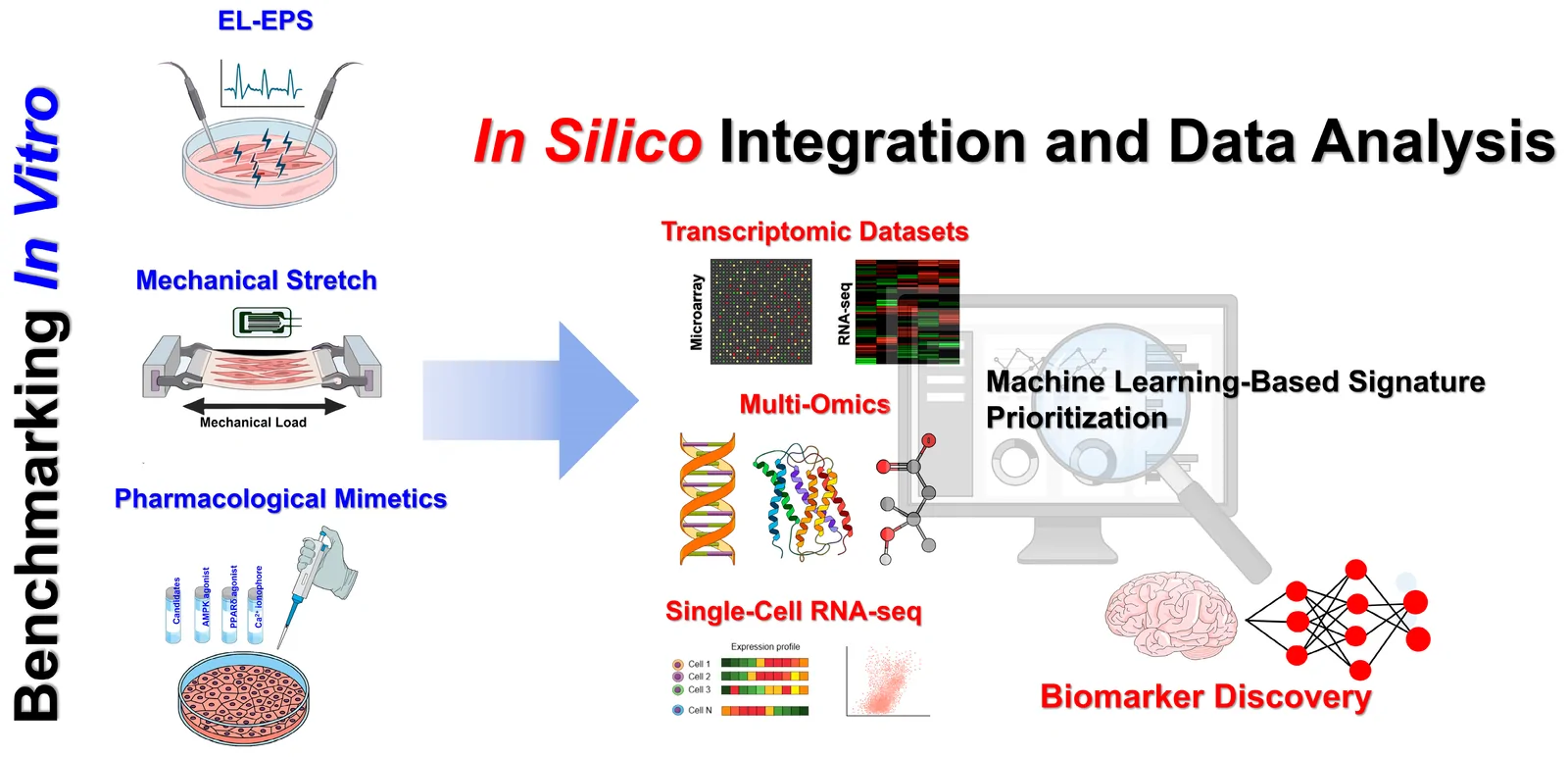
Conceptual framework integrating in vitro models of exercise-associated adaptations with in silico computational pipelines. Reductionist approaches, including exercise-like electrical pulse stimulation (EL-EPS), mechanical stretch, and pharmacological exercise mimetics, generate controlled biological samples for transcriptomic and multi-omics profiling. Cross-dataset benchmarking, machine learning, and single-cell or cell-type-aware analyses can then prioritize conserved exercise-responsive signals and candidate biomarkers. These outputs should be considered hypothesis-generating until they are independently replicated and functionally validated in sarcopenia-relevant experimental or clinical settings.

**Table 2:** Comparative characteristics of in vitro approaches for modeling selected exercise-associated adaptations.

Approach/Model	Biological Input or Method	Principal Responses and Recommended Endpoints	Strengths	Limitations and Interpretation	Key References
2D EL-EPS in differentiated C2C12, L6, or primary human myotubes	Programmable electrical pulses inducing repeated membrane depolarization, Ca^2+^ cycling, ATP turnover, and contractile activity	Contractile activity; Ca^2+^ cycling; AMPK signaling; glucose uptake; fatty acid oxidation; mitochondrial and contraction-responsive transcriptional programs	Provides a controlled, cell-autonomous contractile input; scalable and compatible with metabolic assays and multi-omics analyses	Responses depend strongly on voltage, pulse width, frequency, duty cycle, and stimulation duration; lacks tissue architecture, passive tension, vascular perfusion, neural input, and systemic regulation; electrode-associated stress and cell damage should be monitored	[61,62,63,64]
EL-EPS in 3D engineered muscle tissues	Electrical stimulation of aligned and anchored skeletal muscle constructs	Twitch and tetanic force; fatigue-like responses; Ca^2+^ handling; sarcomeric maturation; myotube hypertrophy; metabolic flux	Links molecular and metabolic responses to measurable contractile function in a tissue-like architecture	Construct maturity, matrix composition, electrode geometry, stimulation regimen, and force-normalization methods vary among laboratories; systemic and neurovascular regulation remains absent	[30,65]
Mechanical stretch in 2D myotubes or 3D engineered muscle tissues	Static or cyclic strain with defined amplitude, frequency, direction, and duration	Focal-adhesion and cytoskeletal signaling; MAPK and PI3K–Akt–mTORC1 signaling; protein synthesis; ECM remodeling; myotube growth and tissue organization	Isolates load-sensitive mechanotransduction and complements contraction-based EL-EPS	Does not reproduce active contraction, neural recruitment, or whole-body resistance exercise; excessive or poorly optimized strain can induce injury, inflammation, or impaired differentiation rather than adaptation	[30,73,74,75,86,87]
AICAR-treated C2C12 myotubes or related skeletal muscle preparations	Cellular uptake of AICAR followed by intracellular conversion to ZMP, producing an AMP-like signal and activating AMPK-associated pathways	AMPK activation; mitochondrial content and respiratory capacity; mitochondrial regulatory programs; glucose uptake and fatty acid oxidation in related skeletal muscle preparations	Provides a controllable means of testing the sufficiency of AMPK-associated energy-sensing pathways, including in models with impaired contractility	Represents predominantly pathway-targeted stimulation; dose- and time-dependent off-target or AMPK-independent effects are possible; does not reproduce Ca^2+^, mechanical, endocrine, immune, or neural inputs	[80,81]
Caffeine- or ionomycin-treated L6 myotubes	Intermittent elevation of cytosolic Ca^2+^ through sarcoplasmic-reticulum Ca^2+^ release or ionophore-mediated Ca^2+^ mobilization	Mitochondrial enzyme expression and mitochondrial biogenesis; Ca^2+^-responsive transcriptional signaling	Separates Ca^2+^-dependent signaling from energetic and mechanical inputs	Continuous or excessive Ca^2+^ elevation can cause myotube loss, mitochondrial stress, protease activation, and cytotoxicity; pharmacological Ca^2+^ mobilization differs from physiological excitation–contraction coupling	[82]
Forskolin-treated C2C12 myotubes	Adenylyl cyclase activation leading to cAMP/PKA/CREB signaling	CREB–PGC-1α interaction; increased *Fndc5* expression; cAMP-responsive transcriptional programs	Provides a controllable method for isolating cAMP-dependent and adrenergic-like signaling	Bypasses adrenergic receptors and systemic catecholamine regulation; pathway activation alone does not establish equivalence to exercise or demonstrate a protective effect	[84]
PPARδ agonist-treated skeletal muscle cells, including GW501516-treated C2C12 cells	Selective activation of PPARδ-dependent transcription	Expression of genes involved in fatty acid utilization, β-oxidation, cholesterol efflux, and energy uncoupling	Enables direct investigation of PPARδ-dependent oxidative and lipid-metabolic programs	Represents single-receptor activation; transcriptional responses do not demonstrate equivalence to whole-body exercise; compound-, dose-, and cell-model-specific effects require appropriate controls	[79,85]

Abbreviations: 2D, two-dimensional; 3D, three-dimensional; AICAR, 5-aminoimidazole-4-carboxamide ribonucleoside; AMPK, adenosine monophosphate-activated protein kinase; cAMP, cyclic adenosine monophosphate; CREB, cAMP response element-binding protein; ECM, extracellular matrix; EL-EPS, exercise-like electrical pulse stimulation; MAPK, mitogen-activated protein kinase; mTORC1, mechanistic target of rapamycin complex 1; PGC-1α, peroxisome proliferator-activated receptor gamma coactivator 1-alpha; PI3K, phosphoinositide 3-kinase; PKA, protein kinase A; PPARδ, peroxisome proliferator-activated receptor delta; ZMP, 5-aminoimidazole-4-carboxamide ribonucleotide. These approaches reproduce selected contraction-, load-, or pathway-associated responses and should not be interpreted as complete in vitro equivalents of whole-body exercise.

## Data Availability

No new data were created or analyzed in this study. Data sharing is not applicable to this article.

## Abbreviations

4E-BP1	Eukaryotic translation initiation factor 4E-binding protein 1
AICAR	5-aminoimidazole-4-carboxamide ribonucleoside
Akt	Protein kinase B
AMPK	Adenosine monophosphate-activated protein kinase
AREG	Amphiregulin
*ATP1B4*	ATPase Na^+^/K^+^ transporting subunit beta 4
CaMK	Ca^2+^/calmodulin-dependent protein kinase
cAMP	Cyclic adenosine monophosphate
DBN	Deep Belief Network
dECM	Decellularized extracellular matrix
*DMRT2*	Doublesex and mab-3 related transcription factor 2
EGFR	Epidermal growth factor receptor
EL-EPS	Exercise-like electrical pulse stimulation
EMT	Engineered muscle tissue
EV	Extracellular vesicle
GEO	Gene Expression Omnibus
GOx	Glucose oxidase
GSE	GEO Series
HRH	Hydrogen peroxide-releasing hydrogel
hPSC	Human pluripotent stem cell
HTS	High-throughput screening
IGF-1	Insulin-like growth factor-1
IL	Interleukin
iPSC	Induced pluripotent stem cell
MAPK	Mitogen-activated protein kinase
mTOR	Mechanistic target of rapamycin
mTORC1	Mechanistic target of rapamycin complex 1
MuSiC	Multi-subject single-cell deconvolution
NCBI	National Center for Biotechnology Information
NMJ	Neuromuscular junction
p70S6K	70 kDa ribosomal protein S6 kinase
PGC-1α	Peroxisome proliferator-activated receptor gamma coactivator 1-alpha
PI3K	Phosphoinositide 3-kinase
PKA	Protein kinase A
PPARδ	Peroxisome proliferator-activated receptor delta
*PPARGC1A*	Peroxisome proliferator-activated receptor gamma coactivator 1-alpha
*PRND*	Prion protein 2
RNA-seq	RNA sequencing
ROS	Reactive oxygen species
*RPS6KB1*	Ribosomal protein S6 kinase B1
SA-β-gal	Senescence-associated β-galactosidase
SASP	Senescence-associated secretory phenotype
scRNA-seq	Single-cell RNA sequencing
snRNA-seq	Single-nucleus RNA sequencing
TNF-α	Tumor necrosis factor-alpha
TWEAK	Tumor necrosis factor-like weak inducer of apoptosis
*USP54*	Ubiquitin-specific peptidase 54
ZMP	5-aminoimidazole-4-carboxamide ribonucleotide
